# ﻿Revisiting the infrageneric classification of *Garcinia* L. (Clusiaceae): an updated sectional name and new, legitimate names in *Garcinia* for *Allanblackiagabonensis* (Pellegr.) Bamps and *A.parviflora* A.Chev.

**DOI:** 10.3897/phytokeys.243.124751

**Published:** 2024-06-20

**Authors:** Patrick Sweeney, Myriam Gaudeul

**Affiliations:** 1 Yale Peabody Museum, Yale University, 170 Whitney Avenue, New Haven, CT 06511, USA Yale University New Haven United States of America; 2 Institut de Systématique, Evolution, Biodiversité (ISYEB), Muséum National d’Histoire Naturelle-CNRS-SU-EPHE-UA, 57 rue Cuvier, CP 39, 75231 Paris, Cedex 05, France Muséum National d’Histoire Naturelle Paris France

**Keywords:** Homonym, nomenclature, priority, taxonomy

## Abstract

In a recent publication dealing with the sectional-level taxonomy of *Garcinia*, an illegitimate superfluous sectional name and two illegitimate homonyms were published. Herein we choose a legitimate sectional name, GarciniasectionRheediopsis Pierre, for the superfluous name GarciniasectionRheedia (L.) S.W.Jones ex P.W.Sweeney; and create two new legitimate names in *Garcinia* for *Allanblackiagabonensis* (Pellegr.) Bamps and *A.parviflora* A.Chev.

## ﻿Introduction

In Gaudeul et al. (2024), Garciniasect.Rheedia (L.) S.W.Jones ex P.W.Sweeney was recognized to include species placed by Jones (1980) into G.sect.Rheedia (L.) S.W.Jones *nom. inval.*, G.sect.Rheediopsis Pierre, and G.sect.Teracentrum Pierre. However, GarciniasectionRheedia (L.) S.W.Jones ex P.W.Sweeney is an illegitimate name (superfluous as per Art. 52.1, Turland et al. 2018) due to the two previously published sectional names being included within it. Instead, we here choose the name GarciniasectionRheediopsis Pierre for this section.

In Gaudeul et al. (2024), the genus *Allanblackia* was placed into synonymy under GarciniasectionAllanblackia (Oliv.) P.W. Sweeney and new combinations and names were created in *Garcinia* for the former *Allanblackia* species. Two of these new names are illegitimate later homonyms (Art. 53.1 Turland et al. 2018). Here we provide new legitimate names for these species.

## ﻿Nomenclature

### ﻿ Garcinia section Rheediopsis

Taxon classificationPlantaeMalpighialesClusiaceae

Pierre, Fl. Forest. Cochinch. 1, Fasc. 5 (1883). Clade 2 in Gaudeul et al. (2024).

DEFE2FDD-5354-5625-A380-0D54194C96F7

#### Type.

*Garciniasmeathmannii* (Planch. & Triana) Oliv., Fl. Trop. Afr. 1: 168 (1868), designated by Gaudeul et al. (2024).

#### Synonyms.

*Rheedia* L., Sp. Pl.: 1193 (1753).

GarciniasectionTeracentrum Pierre, Fl. Forest. Cochinch. 1, Fasc. 5 (1883).

GarciniasectionRheedia (L.) S.W.Jones ex P.W.Sweeney, *nom. illegit*., PhytoKeys 239: 86 (2024).

#### Species.

*Garciniaalbuquerquei* (M.E.Berg) Bittrich; *G.ambrensis* (H.Perrier) P.W.Sweeney & Z.S.Rogers; *G.anjouanensis* (H.Perrier) P.W.Sweeney & Z.S.Rogers; *G.aphanophlebia* Baker; *G.apostoloi* Mouzinho; *G.arenicola* (Jum. & H.Perrier) P.W.Sweeney & Z.S.Rogers; *G.aristata* (Griseb.) Borhidi; *G.bakeriana* (Urb.) Borhidi; *G.barkeriana* (Urb. & Ekman) Alain; *G.benthamiana* (Planch. & Triana) Pipoly; *G.brasiliensis* Mart.; *G.calcicola* (Jum. & H.Perrier) P.W.Sweeney & Z.S.Rogers; *G.cincta* (Urb.) Borhidi; *G.clarensis* Borhidi; *G.commersonii* (Planch. & Triana) Vesque; *G.cubensis* (Borhidi) Borhidi; *G.dalleizettei* (H.Perrier) P.W.Sweeney & Z.S.Rogers; *G.decussata* C.D.Adams; *G.floribunda* Miq.; *G.fluviatilis* Mouzinho & L.Marinho; *G.gabonensis* Sosef & Dauby; *G.gardneriana* (Planch. & Triana) Zappi; G.×guacopary (S.Moore) M.Nee; *G.hessii* (Britton) Alain; *G.humilis* (Vahl) C.D.Adams; *G.intermedia* (Pittier) Hammel; *G.kingaensis* Engl.; *G.leptophylla* Bittrich; *G.livingstonei* T.Anderson; *G.macrophylla* Mart.; *G.madruno* (Kunth) Hammel; *G.magnifolia* (Pittier) Hammel; *G.magnophylla* (Cuatrec.) Hammel; *G.mangorensis* (R.Vig. & Humbert) P.W.Sweeney & Z.S.Rogers; *G.martinii* (Maguire) Govaerts; *G.megistophylla* P.W.Sweeney & Z.S.Rogers; *G.moaensis* (Bisse) Borhidi; *G.obliqua* Sosef & Dauby; *G.ophiticola* (Borhidi) Borhidi; *G.ovalifolia* Oliv.; *G.pachyclada* N.Robson; *G.parviflora* Benth.; *G.pervillei* (Planch. & Triana) Vesque; *G.polyneura* (Urb.) Borhidi; *G.portoricensis* (Urb.) Alain; *G.pulvinata* (Planch. & Triana) Hammel; *G.pungens* Borhidi; *G.revoluta* (Urb.) Borhidi; *G.robsoniana* Bamps; *G.ruscifolia* (Griseb.) Borhidi; *G.semseii* Verdc.; *G.serpentini* Borhidi; *G.smeathmannii* (Planch. & Triana) Oliv.; *G.spruceana* (Engl.) Mouzinho; *G.staudtii* Engl.; *G.thouvenotii* (H.Perrier) P.W.Sweeney & Z.S.Rogers; *G.tsimatimia* P.W.Sweeney & Z.S.Rogers; *G.urschii* (H.Perrier) P.W.Sweeney & Z.S.Rogers; *G.verticillata* Alain.

##### ﻿New names for species included in Garciniasect.Allanblackia (Oliv.) P.W. Sweeney

### ﻿ Garcinia alepensis

Taxon classificationPlantaeMalpighialesClusiaceae

P.W.Sweeney nom. nov.

0E724198-E97E-5ABF-9F7A-81005D9511D5

urn:lsid:ipni.org:names:77339326-1

≡Allanblackiaparviflora A.Chev., Vég. Ut. Afr. Trop. Franç. 5: 163 (1909). Type. Côte d’Ivoire: Alépé, *Chevalier 16239*. non Garciniaparviflora Benth., London J. Bot. 2: 370 (1843).
≡Garciniaguineensis P.W.Sweeney, PhytoKeys 239: 87 (2024), *nom. illeg.* non Garciniaguineensis (G.Don) Vesque, Monogr. Phan. [A.DC. & C.DC.] 8: 335 (1893).

#### Note.

A replacement name, *Garciniaalepensis*, is created here for *Allanblackiaparviflora*, because the epithet *parviflora* was used previously in *Garcinia* for a different species. The epithet *alepensis* is chosen in reference to the type locality of the species, Alepe, Côte d’Ivoire.

### ﻿ Garcinia agnoume

Taxon classificationPlantaeMalpighialesClusiaceae

P.W.Sweeney nom. nov.

73104F77-8A62-50FA-8645-0B4B6753AF9E

urn:lsid:ipni.org:names:77341601-1

≡Allanblackiagabonensis (Pellegr.) Bamps, Bull. Jard. Bot. Natl. Belg. 39: 356 (1969). Type. Gabon: between Moubighou and Nzoundou, *Le Testu 6001*. non Garciniagabonensis Sosef & Dauby, PhytoKeys 17: 52 (2012).
≡Garciniangouniensis P.W.Sweeney, PhytoKeys 239: 88 (2024), *nom. illeg.*
non Garciniangouniensis Pellegr., Bull. Mus. Natl. Hist. Nat. 27: 195 (1921).

#### Note.

A replacement name, *Garciniaagnoume*, is created here for *Allanblackiagabonensis*, because the epithet *gabonensis* was used previously in *Garcinia* for a different species. The epithet *agnoume*, constructed as a noun in apposition, is in reference to a vernacular name *agnoumé* used for this species within parts of its range (Eyog Matig et al. 2006).

## Supplementary Material

XML Treatment for Garcinia section Rheediopsis

XML Treatment for Garcinia alepensis

XML Treatment for Garcinia agnoume

## ﻿Additional information

### Author ORCIDs

Patrick Sweeney https://orcid.org/0000-0003-1239-189X

### Data availability

All of the data that support the findings of this study are available in the main text.
